# Optogenetic Stimulation in a Computational Model of the Basal Ganglia Biases Action Selection and Reward Prediction Error

**DOI:** 10.1371/journal.pone.0090578

**Published:** 2014-03-10

**Authors:** Pierre Berthet, Anders Lansner

**Affiliations:** 1 Numerical Analysis and Computer Science, Stockholm University, Stockholm, Sweden; 2 Department of Computational Biology, School of Computer Science and Communication, KTH Royal Institute of Technology, Stockholm, Sweden; 3 Stockholm Brain Institute, Karolinska Institute, Stockholm, Sweden; The University of Queensland, Australia

## Abstract

Optogenetic stimulation of specific types of medium spiny neurons (MSNs) in the striatum has been shown to bias the selection of mice in a two choices task. This shift is dependent on the localisation and on the intensity of the stimulation but also on the recent reward history. We have implemented a way to simulate this increased activity produced by the optical flash in our computational model of the basal ganglia (BG). This abstract model features the direct and indirect pathways commonly described in biology, and a reward prediction pathway (RP). The framework is similar to Actor-Critic methods and to the ventral/dorsal distinction in the striatum. We thus investigated the impact on the selection caused by an added stimulation in each of the three pathways. We were able to reproduce in our model the bias in action selection observed in mice. Our results also showed that biasing the reward prediction is sufficient to create a modification in the action selection. However, we had to increase the percentage of trials with stimulation relative to that in experiments in order to impact the selection. We found that increasing only the reward prediction had a different effect if the stimulation in RP was action dependent (only for a specific action) or not. We further looked at the evolution of the change in the weights depending on the stage of learning within a block. A bias in RP impacts the plasticity differently depending on that stage but also on the outcome. It remains to experimentally test how the dopaminergic neurons are affected by specific stimulations of neurons in the striatum and to relate data to predictions of our model.

## Introduction

In situations where multiple choices are available, selection might rely on the relative estimated value of each possible action. The one with the highest value, i.e. greatest expected return, should thus be more likely selected. The diversity of the information that basal ganglia (BG) receive, their functional architecture and their learning properties have brought the BG to be considered as a centralized action selection device, specialized to resolve conflicts over access to limited motor and cognitive control [1] and to analyse the cost-benefit of actions [2]. The BG receive information from various parts of the cortex and the thalamus [3]. They also get connections from amygdala and dopaminergic neurons [4]–[6]. Dopamine level has been shown to be critical in the modulation of the plasticity of the cortico-striatal synapses [7], [8]. Electrophysiological recordings in the striatum have shown that it could encode the representation of action values [9]–[12]. Computational models of the BG based on a three factors update rule have been able to give results similar to experimental data [13]–[15]. The dopamine signal is believed to code the reward prediction error (RPE), i.e. the difference between the expected and the actual reward [16]–[21]. BG also feature a dual pathways architecture that shows complementary functionalities: both pathways stem from GABAergic medium spiny neurons (MSNs) in the striatum but differ with respect to the dopamine receptor these MSNs express [22]. The D1 receptor type, giving rise to the “direct”, pathway is believed to promote an action. The D2 type one, from where the “indirect” pathway originates, would be involved in inhibiting actions. Thus, stimulation of a specific pathway can bias the behaviour accordingly [23]–[25]. Optogenetic studies, where a specific type of dopamine receptor expressing MSN was infected with channelrhodopsin 2 (ChR2), have brought support to the dual pathways categorisation. Stimulations of the D1 MSNs in dorso-medial striatum (DMS) have been shown to increase motor activity [23] and the probability of selecting the contra lateral side out of two lateralised options [26], and to reduce Parkinson's disease motor symptoms in animal model [27]. Stimulation of the D2 pathway produces opposite effects. Behavioural modification have also been described with phasic optical activation of dopaminergic neurons in time of, and instead of, the delivery of the actual reward [28]. Striatum and pallidum have been shown to project to SNc and ventral tegmental area (VTA), two main dopaminergic nuclei [29], [30]. However, the simulation of optogenetic activation in computational models has not been well investigated. We implemented in our abstract model of the BG the possibility to selectively increase the action value in one of the direct or indirect pathway and also in the reward prediction (RP) system. Furthermore, we aimed to study the possible effects of the stimulation on the plasticity and how the dopaminergic system might be impacted. We compared our results to experimental data from Tai & Lee et al. [26] on mice. We also tested the implication of the localisation of the stimulation. We then discuss the possible causes and consequences on the reward prediction of stimulations in striatum, based on the results of the model.

## Results

We used a network consisting of five states, i.e. cortical activation patterns, and four actions, with two of the latter arbitrarily designed as “right” or “left” choice. All states were activated during a simulation but we recorded only the trials relative to one pre-defined state. The system was constrained to select either one of these two actions during the task. In our model, all the different states project via both the D1 and the D2 pathways to all the actions and the connections are all subject to the same rule (see *Material and Methods*). With respect to the Actor-Critic framework [31], these two pathways constitute the Actor part of the model and are directly involved in the action selection. It has been shown that direct and indirect pathway spiny projection neurons (SPNs) in striatum respond to both intra-telencephalic and pyramidal tract activation [32]. Our model uses a complementary activation pattern in the two pathways. The D1 pathway promotes one action while the D2 can suppress all the other possible actions in order to get a unique well defined action performed. The role of the Critic would be filled by the RP system, which informs the Actor of the difference between the expected outcome and its actual value. RP computes the reward prediction for all the possible state-action combinations. Units in RP receive inhibitory plastic projections from the states and the actions layers. Delivery of a reward is equivalent to an increase in the value of the relevant RP unit. The prediction is specific to a state-action pairing. RP thus needs to know the current state and the selected action. This occurs once the selection has been made, but before the system receives information about the outcome. The RPE is thus the difference between the expected reward for the current state-action pairing and the actual reward. RPE is then sent from RP to the states-actions connections in the direct and indirect pathways, and also to projections from the states and the actions to RP. This enables RP to learn to predict the correct reward value. For example, if the reward is larger than expected, the weights from the current state and the selected action to RP will be increased. Thus, at the next occurrence of that situation, units in RP will be more inhibited. Hence, for the same actual reward value, the RPE will be smaller. Depending on the pathway and the sign of the RPE, the updates triggers opposite effects. A trial, equivalent to updating the model by one time step, occurs, in summary, as follows:

- activation of a unique unit in the state (cortical) layer,
- computation of the activation of the units in the action layer (BG) and selection of one of these actions via the softmax function (see *Materials and Methods*),
- computation of the RP based on the action selected and the current state,
- “performing” the action and receiving a reward value from outside the system,
- computation of the RPE and finally updating the weights and biases in the network.

In their study, Tai & Lee et al. [26] selectively injected a virus into the dorsomedial striatum (DMS) of mice that enabled expression of ChR2 in either D1 or D2 MSNs. They could thus give transient uni-lateral stimulations, via optical flashes, to activate specific MSNs populations, in a two choices, “left” or “right”, task. These stimulations lead to an increase in the firing rate of the targeted infected neurons on par with the flashes. The results from stimulations at three different intensities are presented in their study. D1 (D2) MSNs stimulations delivered coincidentally with the onset of the cue increased (decreased) the probability of selecting the contra-lateral side. Furthermore, the extent of this bias correlated with the intensity of the stimulation but depended also on the previous reward history. The largest effect was recorded when there had been inconsistency in the recent reward delivery.

It is however unknown how the increased activity of the targeted MSNs during an optical stimulation leads to the bias in action selection and how the neural activity of connected structures is affected. In order to detail the functional and anatomical connections between striatum and SNc, we investigated how the model would be impacted by the stimulation. We implemented the possibility to increase the action value of one unit in the direct or the indirect pathway for one trial (see *Materials and Methods*). Specifically, we also varied the impact on the RP pathway. Striosomal MSNs have been shown to project mainly to SNc [33], [34], which is commonly believed to be involved in coding the RPE [18], [35]. There are also inhibitory connections from GPi/SNr to SNc. It could thus be possible that the optical stimulation of the striatal MSNs indirectly modifies the RP system, supposedly by increasing (decreasing) the predicted reward value of the action contra-lateral of the stimulation for D1 (D2) ChR2 affected MSNs. This could in turn affect the RPE and eventually the modification of the weights. All in all, this could indirectly affect subsequent action selections.

To test whether or not the increased activity of the ChR2 MSNs during an optical flash also affects the reward prediction, we first compared our model in two conditions. In the first one, the added action value only affected the relative action value in its specific pathway D1 or D2. In the second one, the relative reward prediction for this action was also impacted accordingly. This did not additionally affect the current selection as it modified the amplitude of the RPE. That also determined the amplitude of the weight updates. The bias that was added in RP had the same value as the one added in the Actor pathway. We had two conditions for the biasing in RP: either RPE was biased independently of the action selected; or it was modified only if the action selected was the one positively biased by the stimulation. The underlying question is whether or not the reward prediction depends on the whole striatal activity or if it is restrained to the activity of the MSNs coding for the selected action. For example, let us take the situation following a stimulation of right hemisphere D1 MSNs, thus increasing the probability of selecting the left side. Is the reward prediction for this trial impacted by this stimulation if the side eventually selected is the other one, that is, the left side? Or is it only biased when the action selected was the one coded by the stimulated MSNs? We thus compared these two options to the basic set up without added bias in RP. Furthermore, we tested the hypothesis that if an increased activity in the D1 MSNs leads to a higher reward prediction in SNc, then performance between trials with and without stimulation should be different depending on the learning stage. During the early phase of learning, a larger than normal, i.e. artificially increased, reward prediction might slow down learning when a reward is actually delivered. The difference between the expected value and the actual reward being smaller, triggering a smaller change in the weight than without the modification of the activity. On the contrary, if no reward is delivered, the decrease in the weight would be larger for trials where a stimulation of D1 MSNs occurred as the difference between the expected reward and the actual reward is larger than in trials without stimulation.

### Simulation of optical stimulations

**Figure 1:**
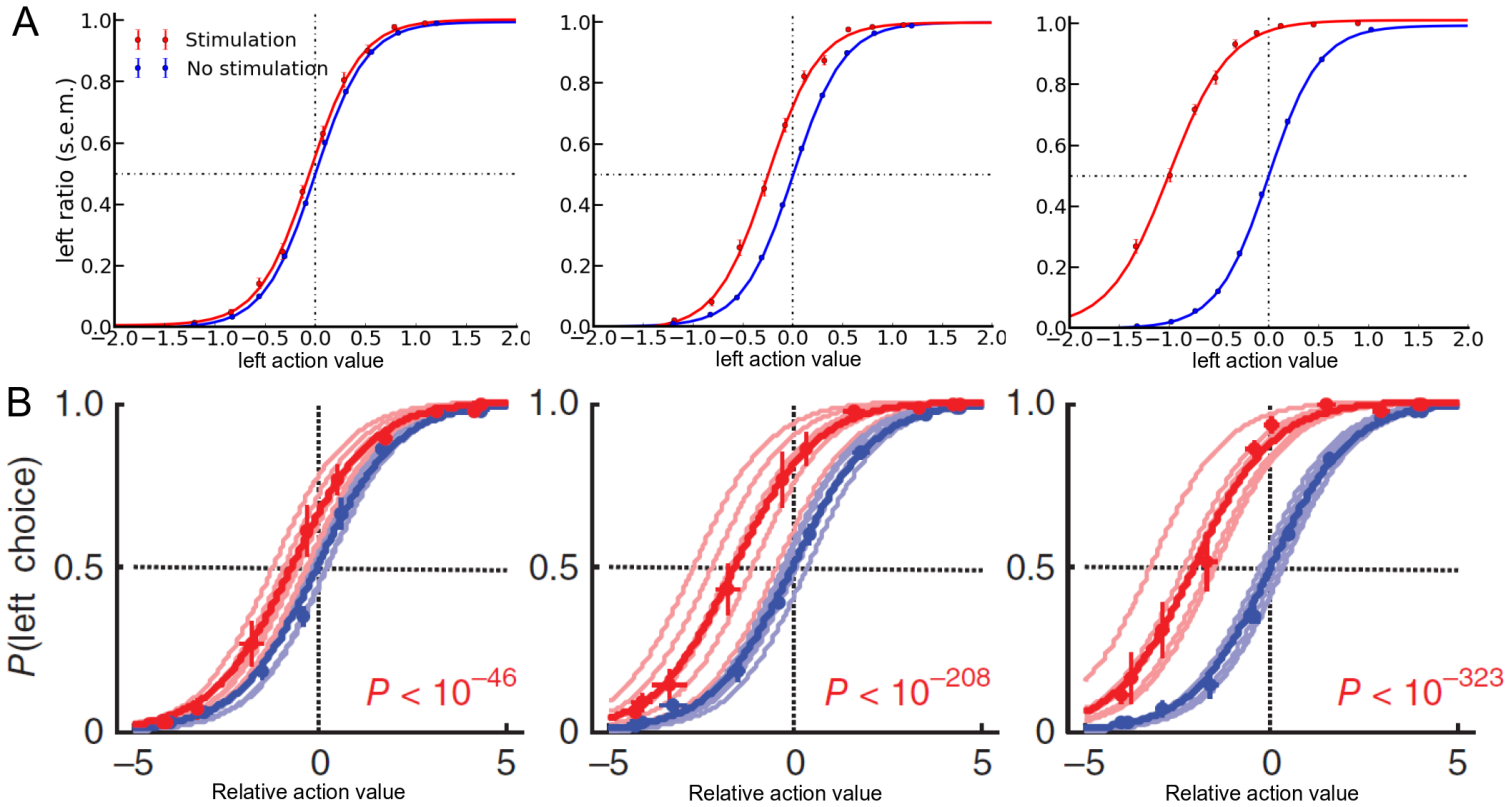
Ratio of left action selection relatively to ten intervals averages of the left action value, without and with added stimulation. Top row (**A**) shows modelling results for three different intensities: 0.15, 0.50 and 2.00, bottom row (**B**) is reproduced from Tai & Lee et al. (2012) with also three different intensities. All error bars represent s.e.m.

The task of the mice was to select one exit out of two possible: “left” and “right”. The exit rewarded was periodically changed at the end of every block of 20 trials. Only one side at a time had a reward probability of 75%, whereas no reward was delivered in the other 25% trials. In our model setup, we had four different conditions in this first tuning part as to where the activity was increased: the two actions, “left” and “right”, in each of the two pathways, D1 and D2. We thus named D1L and D1R a localisation of the stimulation in the D1 pathway for action “right” and “left” respectively (to reproduce the contra-lateralisation), also D2L and D2R denote a similar notation in the D2 pathway. We tested different values for the added activity in order to test if the model could reproduce the biases in the action selection. In 6% of the trials, the value relative to the contralateral action in a specific pathway was increased just before the softmax selection process. The location and intensity of this added activity were kept constant within a simulation of 4000 trials. For this validation phase, we used a network with only two actions, arbitrarily labelled “left” and “right”. With this simple setup, we found three values: 0.15, 0.50 and 2.0, that showed a significant bias in the action selection, similar to the experimental results. Figure 1 shows the results for the three different stimulation value for D1R localisation. As expected, and similar to what happened in the experimental study [26], the probability of selection of an action increased as its relative action value increased (p<0.001). Our results were also similar to the experimental observations for the other stimulation localisations: D1L, D2L and D2R (not shown).

**Figure 2:**
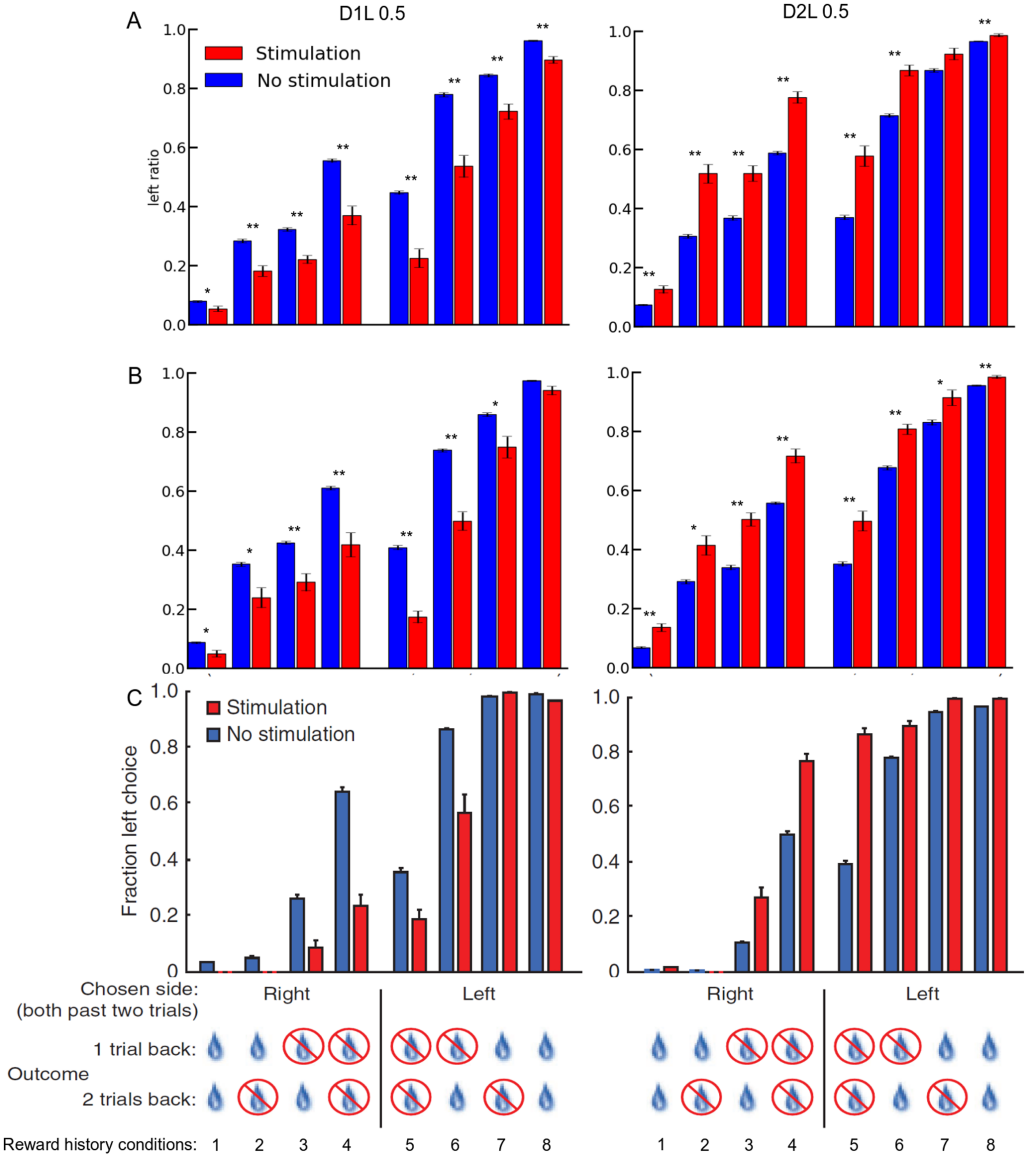
Ratio of left action selection relatively to the two previous trials history for D1L (left column) and D2L (right column) stimulation. **A** and **B** show our modelling results and **C** reproduces results from Tai & Lee et al. (2012). RP was not affected by the stimulation in **A**, whereas it was the case in **B**, but only if the selected action was the one contralateral to the stimulation. All error bars represent s.e.m., * and ** means p<0.05 and p<0.001 respectively for the difference in left ratio with and without stimulation, for each reward history condition.

We then took into account the recent reward history. This enabled us to test whether the bias caused by the stimulation depends on the previous trials and their outcome. Such bias had been observed in mice [26]. Specifically, we looked at the action selection distributions when the same action was selected for the previous two trials. We recorded the associated outcomes for these previous trials. Thus, this gives eight cases (figure 2) for each of the two stimulation conditions, i.e. with and without added stimulation. We used an intensity of 0.5 as the stimulation value added to the action value, either in D1L or D2L. Figure 2 shows the proportion of “left” action selected relatively to the different cases, both for trials with and without stimulation. We also ran a version of the test in which the RP pathway was biased, with respect to the stimulation localisation (figure 2B). We modified the value of the unit in RP coding for the reward prediction of the current state-action pairing. The amplitude of this stimulation was the same as the one used in the direct or indirect pathway. However, this was not done systematically. In order to investigate the impact on the selection that a stimulation of RP could have, we defined two conditions where the stimulation could also modify RP. We could either change the RPE in all the trials with stimulation, or be more restrictive and only change the RPE if the action selected was the one contra-lateral to the stimulation. The underlying concept here is about the functional connection between striatum and dopaminergic neurons coding for the RPE. It is possible that the reward prediction depends on the global activity of the MSNs in striatum. It might also be that it depends specifically on the activity of the MSNs coding for the action that has been selected. Furthermore, we differentiated stimulations in the direct and the indirect pathway. Stimulation of the direct pathway led to an increase of the relative reward prediction by inhibiting the RP units more. Stimulation of the indirect pathway led to a decrease in the reward prediction, down to zero. We suggest that inhibitory connections from D2 MSNs could target interneurons in SNc instead of projecting directly to dopaminergic neurons. This would enable an increase in the dopaminergic neurons firing rate by inhibition of their inhibitory interneurons.

We averaged the probability distribution of the selection of action “left” over multiple runs of the simulation. We performed Student's T-tests on these results to compare selection ratio for the two stimulation conditions on each different history. Significant differences were seen predominantly for the trials where the positive reward delivery had been inconsistent or absent in the two previous trials. These differences were less significant when rewards were consistently delivered during this period. These results show great similarities with the experimental results of Tai & Lee et al. [26], both for the conditions with and without stimulation and for the different localisations of the stimulation.

We then focused on the RP pathway and the impact that RP-specific stimulation could have on the selection. Globally, the alteration of RP did not dramatically change the profile. More specifically, it seems to have decreased the effect of the stimulation. There was no difference in the selection ratio between the two conditions of inclusion of RP in the biasing (action dependent versus action independent, results not shown), or between the results of the different simulations without stimulation. All in all, the results of simulations where RP is impacted seem to be closer to the experimental results. The latter did not show a significant effect of the stimulation when a reward was delivered, or omitted, consecutively. The results of simulations with RP stimulation show a decrease in the significance for these same conditions, compared to the results of simulations without RP stimulation.

**Figure 3:**
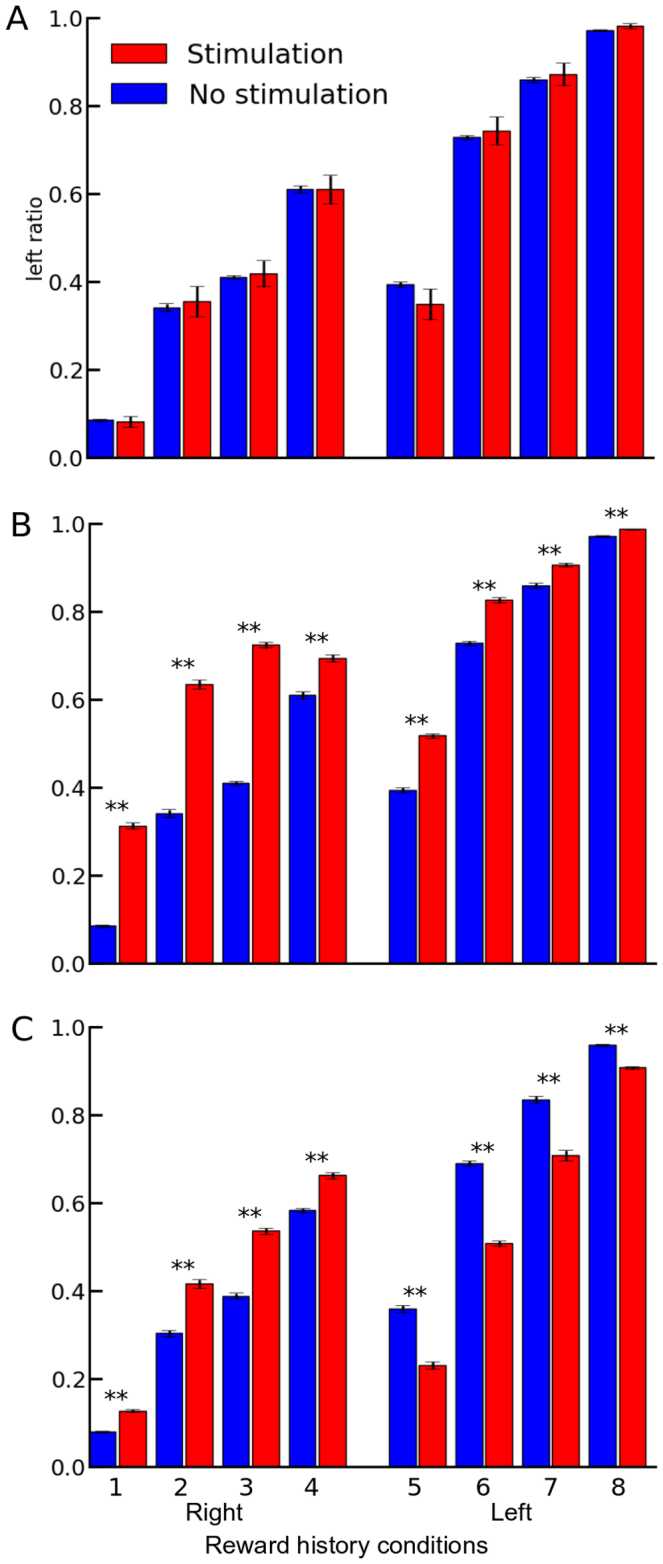
Impact on the action selection of an added activation (+0.5) only in the reward prediction. Results represented in blue come for simulations without any stimulation. In red, in **A** and **B**, the reward prediction was increased only if the action selected was “right”. **A** 6% of the trials could lead to a stimulation, whereas in **B**, this stimulation could occur in 94% of the trials. In **C**, the reward prediction was modified independently of the action selected, in 94% of the trials. **represents a significant difference (p<0.001) between conditions with and without stimulation for the same reward history. The reward history conditions are similar to figure 3. Error bars represent s.e.m.

We then proceeded to probe how the system would be affected when only the reward prediction was targeted by the stimulation, i.e. without biasing the D1 or D2 pathway. We also looked at how the dependence on the action selected would modify the selection profile. Relatively to the Actor-Critic framework, ventral stimulation of the striatum could result in a predominant alteration of the dopaminergic signalling, here represented by the RP pathway, over the direct and indirect pathways. This could impact the plasticity, via a modification of the RPE. We chose to use the same set-up as previously to facilitate comparisons. From here on, the stimulation has the same value for the subsequent tests: 0.5. Figure 3 presents the results for stimulation involving the RPE, still occurring with a 6% probability (figure 3A). However, as the RPE is computed after the selection, any potential effect can only be noticed in subsequent trials. We thus did not expect that the stimulation of only the RPE would induce a shift. In conformity with the previous results, with a probability of stimulation of only 6%, there is no difference with the selection profile from a simulation without stimulation (figure 3A). Any specific effect due to the bias is probably flooded amongst all the trials without stimulation. We thus compared these results to two additional conditions: one where stimulations were delivered in 94% of the trials, in which the RPE would effectively be biased only if the action selected was “right” (figure 3B) and another one where the reward prediction was biased in 94% of the trials, independently of the action selected (figure 3C). In that case, the results indicate a strong bias (all differences p<0.001) in the selection towards the “left” action for both the action-dependent and action-independent conditions when the two previous actions selected were “right”. Interestingly, these two conditions show opposite bias in the selection ratio when the two previous actions selected were “left”. The action-dependent condition still exhibits a bias towards a “left” selection compared to the simulation without stimulation, although weaker than for the trials where the two previous actions selected were “right”. The action-independent condition for the “right” action history now exhibits a large decrease in the “left” selection ratio compared to the simulation without stimulation.

**Figure 4:**
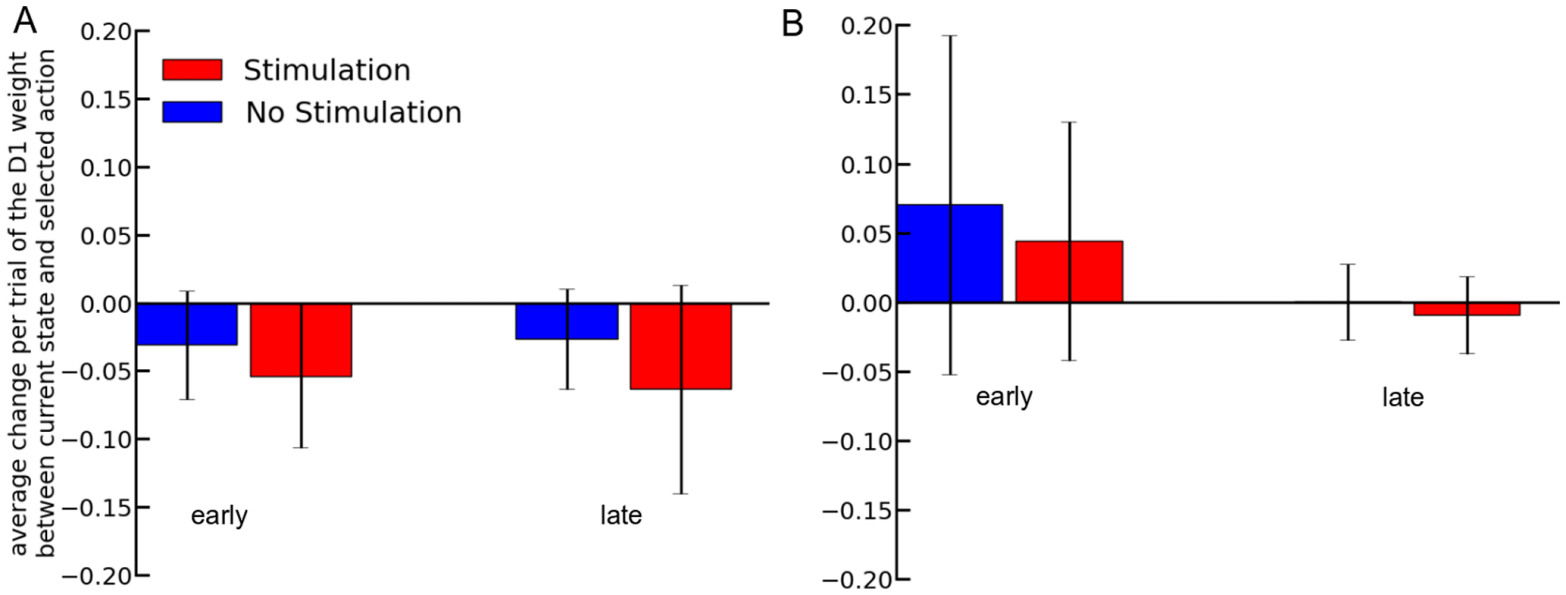
Average change in the weights at the beginning (first two trials) and at the end (last two trials) of blocks of 20 trials. Results group trials where no reward was delivered are presented in **A**, whereas in **B**, a reward was obtained. Error bars represent standard deviation and all differences for a same timing are significant (p<0.001).

We then looked more precisely at the impact on the change of the weights caused by a bias of the RPE (figure 4). We compared the average change of the weights at the beginning (first two trials) and at the end (last two trials) of a block of 20 trials from a simulation of 5000 blocks. The probability of biasing the RPE was 15%. In that event, the reward prediction was increased by 0.5, the same value used in the previous tests. We recorded only the change of the weights of the connection between the active state and the selected action, in the D1 pathway. As there were two outcomes for every trial, i.e. the delivery of a reward or not, we thus divided the results to take into account these two different possibilities. We ran a Two-Way ANOVA on the average change of the weights in the different conditions. It showed a significant effect of the stimulation (figure 4, blue versus red), of the outcome (figure 4, A versus B), of the position of the trial in the block (figure 4, early versus late), and of their multiple interactions, on the average change of the weights (p<0.001).

For trials without reward, the larger expectation created by the stimulation produces a larger, compared to the change in trials without stimulation, decrease of the weights of the action selected in the direct pathway. The difference for a same position within a block, i.e. early or late, is significant between the two stimulation conditions. However, when there is no reward delivered, there is no significant difference in the change of the weights between the “early” and “late” stage for a same stimulation condition. In the condition with reward, all the differences are significant (p<0.001).

The change in the weight between the state and the selected action decreases between early and late trials for the two stimulation conditions when a reward was obtained. Also, the difference between the two stimulation conditions is larger for trials at the beginning of a block than at the end.

It has to be noted that if the probability of stimulation is too high, then the system learns to integrate the bias in the prediction of the reward. Thus, for that set-up, there is not any longer a significant difference at the end of a block between a condition with and without stimulation (result not shown).

## Discussion

We were able to reproduce quantitatively in our model the shift in action selection observed in mice subjected to optogenetic stimulation. The stimulation biases the distribution of the action values in striatum. It is when the uncertainty about the expected reward is high, that is when the reward obtained in the two previous trials has been inconsistent, that the impact of the simulated stimulation on the action selection has the largest effect. This result adds support to the view that the softmax selection process used in our model is a good approximation of the mechanisms behind exploratory behaviour [19], [36]. We then investigated how stimulations in different part of the system could impact the selection. Our model enables us to selectively modify the value in each of its pathways. We thus tested the hypothesis that the optogenetic stimulation could impact indirectly, via the increased MSNs activity and their connections to the dopaminergic neurons, the RP system. This would affect the dopamine modulated plasticity of the cortico-striatal connections. We did not observe a large impact on the results by extending the stimulation to RP.

The differences shown in figure 2 between results from our model and from experiments could be explained by plasticity. Plasticity can occur with different time constants. There could be different time constants not only for potentiation and depression, but also depending on the dopamine receptor types of the MSNs. We had one unique time constant for all the updates in the D1 and D2 pathways, but a slower one for the RP pathway.

Also, a distinction is commonly made between the dorsal and the ventral part of the striatum [37]. The former is thought to correspond to the Actor and the latter to the Critic. Dorsal parts of the striatum project mainly to GPe and to GPi/SNr whereas ventral regions connect to dopaminergic neurons of SNc. With respect to this distinction, it is possible that the stimulation of a more ventral part would have had a larger impact on the activity in SNc and thus in the dopamine signal. Scarce RP stimulation didn't have any impact on the selection of the model. This is congruent with experimental results, showing an effect of DMS stimulation [23], [26] but no effect of activation of nucleus accumbens [38]. This supports the idea that the action selection process does not involve the ventral part in mice. It could however impact the plasticity and thus the learning process by biasing the dopamine signal. This would not affect the current selection but would change relatively the probability of selection of subsequent trials.

It has furthermore been suggested that a stimulation of the dorsal part of the striatum might affect the firing of dopaminergic neurons in SNc via inhibitory connections from GPi/SNr. These are the converging nuclei of the direct and indirect pathways [39]. Specifically increasing the activity of a population of MSNs in the striatum might impact the dopaminergic neurons in SNc more than an increased global input from cortex and thalamus. The latter project not only to MSNs but also to fast spiking (FS), tonically active cholinergic neurons (TAN) and low threshold spiking (LTS) interneurons [40], [41]. This architecture might distribute the activity across the striatal network. This would thus prevent an increase restricted to a specific cell type that the optical stimulation produces. The exact role and dynamic of these interneurons, which independently modulate MSNs activity [42], is still unknown in such conditions. It is thus possible that an optogenetic stimulation of a more ventral part of the striatum might produce relatively similar results to a dorsal one, regarding dopaminergic neuron activity.

We investigated if the reward prediction could be simply based on the global striatal activity or if it is affected specifically by the activity of the neurons coding for the selected action. We found no difference when the reward prediction was modified together with the stimulation in the direct or indirect pathway. It is possible that the low probability of occurrence of the stimulations, and the fact that a bias in RP does not interfere with the current action selection, prevent the effect of RP stimulation to be noted. By specifically biasing RP with a very high probability, 94%, a shift in the action selection was observed. For the action-dependent bias of RP, a larger than normal reward expectation for the action “right” lead to an increase in “left” selection for all the reward history conditions. The effect was however smaller when the two previous actions selected were “left” rather than “right”. An explanation for this observation is that the RPE, being modified only when the system selected “right”, is not affected for trials where “left” was selected. However, there is still a significant difference for that side. We suggest that this is indeed also resulting from the modification of the RPE, as it affects the plasticity of all the connections. Such a phenomenon seem to happen also in biology, as cortical connections to D1 and D2 MSNs have been shown to express long term potentiation and long term depression in a broad range of pre- and postsynaptic activity [43]. Previous trials where “right” was selected thus also had an impact on the weights update of all the actions, even small. We have assumed that activity in the indirect pathway leads to a decrease in the associated reward prediction. It remains to experimentally investigate how activity in the direct and indirect pathways affects the reward prediction. It could be that activity in the indirect pathway increases negative reward prediction [44], [45]. It is also possible that the indirect pathway is not at all involved in the reward prediction computation.

For the action independent condition, the shift in the action selection was towards the opposite side relative to those selected for the two previous trials. We suggest that this is caused by the fact that the weight in D1 (D2) between the state and the selected action is more decreased (increased) when the reward is not obtained, due to the higher expected reward value, and thus the larger RPE, than it is increased (decreased) when a reward is delivered, as the RPE is smaller. It should thus be noted that the shift in the selection was larger for trials where the recent reward history shows at least one missed reward. The model is thus less prone to stick with an action that has recently been unrewarded. This is confirmed by the smaller range in the selection ratio for this set up, i.e. it never reaches any of the two extremes (0 or 1).

After unrewarded trials with increased reward prediction, the RPE is larger than it would have been without stimulation. Thus, the weights change is also larger for this trial. Specifically, in the D1 pathway, the weight between the current state and the selected action are more decreased. In the D2 pathway, it is more increased. Furthermore, the opposite happens when a reward is delivered: weights do not change as much as they would have without the extra activation of the reward prediction. All in all, it requires more trials to restore the selection probability of that action to a sufficient, “selectable”, level compared to the condition without stimulation. This could explain the difference between “left” and “right” action history, as the action which was affected by the stimulation was “right”. Recent work using optogenetic techniques to selectively activate dopaminergic neurons in the ventral tegmental area coincidentally with reward delivery has shown that a modification of the RPE is sufficient to impact behaviour in rats [46]. When the probability of stimulation was increased, our model predicted a reward value equal to the sum of the actual reward with the stimulation induced bias. The only information that the system gets is the RPE. It is thus not possible to know if the prediction of the model was artificially increased, or if the actual reward was just smaller.

Regarding the weight evolution, there is no difference in the average weight change when no reward was delivered, for both conditions. This might be due to the fact that, *in fine*, this is a comparison of very similar trials in time and in their outcome. How is it possible to tell the difference between a non-rewarded “correct” trial, because there was 25% chance that a correct choice led to an absence of reward, and a plain wrong trial, where the reward was then associated with the other side because it marked the start of a new block of 20 trials? Similarly, it is not possible to tell if the reward has been smaller, or if the “punishment” has been larger, from the situation where the expectation has been artificially increased before the delivery of a reward, or its absence, respectively.

Our model suggests that a modification of the reward prediction is sufficient to cause a subsequent change in the action selection. It remains to test experimentally how stimulation of MSNs in striatum, especially the ventral part, impact the dopamine signal but we have here suggested two possible profiles of action selection and their functional connectivity implications. It has been suggested that functionally related areas would patch together in striatum [47] and that BG could feature a somatotopic organisation [48].

We didn't investigate the effect of the length of the stimulation, or of its precise timing in the trial, as our model did not implement real time and delays, although timing has been shown to differentially alter reward consumption [49]. A spiking version of the model, currently in development, would enable us to investigate these issues and the impact of the induced activity on reaction times, which were shown to also be affected by the stimulation [26], [50]. It has been observed that DMS is involved in energizing performance vigour [51]. We believe that this could be implemented and that a change of the gain value in the spiking implementation of the softmax function could be a good candidate to take into account the change in reaction time. In our model, the values of the two actions that can be selected would be lowered because of recent less-than-expected reward deliveries. The softmax function would, in that case, require more iterations to pick one of these required actions compared to situations where they would have had a higher value.

Furthermore, a more detailed and comprehensive spiking BG model would enable us to compare the response of the network to deep brain stimulation and to additional forms of optogenetic stimulation. Finally, it has been suggested that BG can receive an efference copy (corollary discharge) of premotor activity [52], notably through thalamus and frontal cortices (see [53], [54]). It remains to investigate if and how this efference copy might be affected by optogenetic stimulation.

## Methods

### Computational model

**Figure 5:**
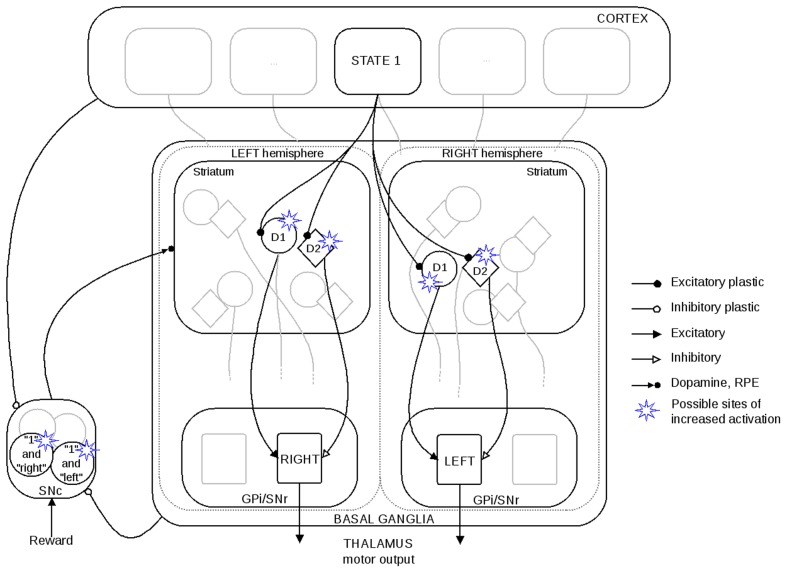
Schematic representation of the model in relation to biology. As not all the different nuclei are implemented in the model, we represented only relevant biological structures. We arbitrarily labelled output actions as “right” and “left”. Only one SNc is shown here, as in our model there is no difference in the feedback (RPE) any D1 or D2 units get. Only one unit in SNc gets active per trial, representing the reward prediction for the current state and the selected action.

Our model is based on BCPNN [55] and is similar to the one in our previous work [56]. It is an abstract model of the BG, where the connections between the units, weights and biases, represent an estimation of the probability of the postsynaptic unit to be active given that the presynaptic unit is active. In this abstract model, an active unit means that the feature it is coding for is currently occurring (figure 5). This can be a state or an action, in a grandmother cell like representation. Thus, sensory evidence can be combined with prior knowledge of the distribution of the events to update ones estimation [57]. This estimation is based on three values: quantification of the activation of the pre- and postsynaptic units, as well as of their co-activation. It is hypothesised that such information can be encoded in connections between neurons. The activation value corresponds to the exponential of the sum of weights and bias of one unit. It has been shown that artificial neural networks and spiking neurons can code Bayesian probabilities [58]–[60]. Cortical inputs are here believed to code the current state and BG represent the different possible actions from which the system has to select one. The strength of the connections between the states and actions in the different pathways is the basis of the selection process.

The model features three pathways which are managed in a similar fashion. The D1 (direct) and the D2 (indirect) pathways are used to compute the action value. The reward prediction system computes the predicted reward based on the current state and the selected action. The basic architecture is similar to other Actor Critic models [31].

The actual selection, based on the activation value that each action unit receives from the D1 and D2 pathways is made by a softmax function. The RPE is then used to update the weights and biases. This signal acts as biasing the probability estimates in order to improve the stimulus-action mapping towards the rewarded pairings. This involves three factors: RPE, pre and post synaptic activity. If the RPE is positive (negative), the main change will occur between the co-active state-action paring in the D1 (D2) pathway. However, if the RPE is negative (positive), the updates will mostly affect the connections between the active state and the non-selected actions in the D1 (D2) pathway. This is similar to what have been observed in biology where the plasticity in D1 and D2 MSNs has shown opposite changes with regard to activity and dopamine level [43], [61]–[63]. Thus when one state is active, the activation in the action layer corresponds to the sum of the activity from the D1 pathway with the negative, inhibitory, activity from the D2 pathway for each action. The activity *s* that a unit *j* in the action layer receives from pathway X is thus defined as
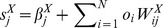
(1)where *o_i_* is 1 for the currently active state unit, and 0 otherwise. The exponential of 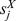 is an estimation of the probability of having action *j* activated while in state *i* in its relative pathway. Thus, the resulting activity in the action layer is
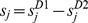
(2)


A softmax activation function, with a gain parameter γ here set to 2, is then applied on these activations to probabilistically select one action.
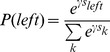
(3)


Based on the current state and the selected action, a reward prediction is made by the RP system. Depending on the current reward mapping, a reward is delivered or not, for this trial. The difference between the actual reward and the predicted value, i.e. the RPE, is then used to update the weights in all the pathways: D1, D2 and RP.

It should be noted that changing the gain parameter γ (here set to 2.0) in the softmax equation (eq. 3) impacted the slope of the sigmoid function, that is, the sharpness of the transition in action selection.

The python source code of the model and instructions can be found as Supporting Information S1.

### Stimulation

To simulate the optically evoked activity, we implemented in our model a function that could increase phasically the activation value of a specific unit in a specific pathway, this unit coding for a single action. Thus, to simulate a left DMS stimulation with D1 MSNs expressing ChR2, we increased the activation in the D1 unit linked with the arbitrarily defined action “right” (figure 5). Similarly, we increased the activation of the D2 unit related with the “left” action to emulate a right DMS optical stimulation of D2 ChR2 MSNs. We also had a condition where we could selectively increase the activation of the reward prediction associated with a specific state-action pairing, thus impacting the RPE. This external activation affected only part of one trial, thus affecting only one specific action selection. The stimulation was added only when either the actual selection was made or, in the case of the RPE, when the update took place. The stimulation value was not included in the action value outside of these events, in order to compare selection ratio for similar action values.

### Statistical analysis

A sigmoid logistic regression using least squares was fitted to the evolution of the left ratio with respect to eleven intervals of the distribution of action values (figure 1).

We performed Student's T-tests on the “left” selection ratio of 20 simulations to compare the conditions with and without stimulation on each different reward history.

We ran a Two-Way ANOVA on the change of the weights. It showed a significant effect of the stimulation (figure 4, blue versus red), of the outcome (figure 4, A versus B), of the position of the trial in the block (figure 4, early versus late), and of their multiple interactions.

## Supporting Information

File S1 **Python source files of the model and instructions on how to run it and to reproduce the results from the study.**
(ZIP)
